# Different Influences of Endometriosis and Pelvic Inflammatory Disease on the Occurrence of Ovarian Cancer

**DOI:** 10.3390/ijerph18168754

**Published:** 2021-08-19

**Authors:** Jing-Yang Huang, Shun-Fa Yang, Pei-Ju Wu, Chun-Hao Wang, Chih-Hsin Tang, Po-Hui Wang

**Affiliations:** 1 Institute of Medicine, Chung Shan Medical University, Taichung 402, Taiwan; cshe961@csh.org.tw (J.-Y.H.); ysf@csmu.edu.tw (S.-F.Y.); cshy1446@csh.org.tw (P.-J.W.); 2 Department of Medical Research, Chung Shan Medical University Hospital, Taichung 402, Taiwan; 3 Department of Obstetrics and Gynecology, Chung Shan Medical University Hospital, Taichung 402, Taiwan; 4 Department of Medicine, National Taiwan University, Taipei 106, Taiwan; cshy222@csh.org.tw; 5 School of Medicine, China Medical University, Taichung 404, Taiwan; chtang@mail.cmu.edu.tw; 6 Chinese Medicine Research Center, China Medical University, Taichung 404, Taiwan; 7 Department of Medical Laboratory Science and Biotechnology, College of Medical and Health Science, Asia University, Taichung 413, Taiwan; 8 School of Medicine, Chung Shan Medical University, Taichung 402, Taiwan

**Keywords:** ovarian cancer, endometriosis, pelvic inflammatory disease, nationwide population cohort, propensity score matching

## Abstract

To compare the rate and risk of ovarian cancer in patients with endometriosis or pelvic inflammatory disease (PID). A nationwide population cohort research compared the risk of ovarian cancer in 135,236 age-matched comparison females, 114,726 PID patients, and 20,510 endometriosis patients out of 982,495 females between 1 January 2002 and 31 December 2014 and ended on the date of confirmation of ovarian cancer, death, or 31 December 2014. In order to reduce the unbalanced characteristics, propensity score matching (PSM) was performed for 20,478 females in each subgroup. The incidence rate (per 100,000 person–years) of ovarian cancer was 8.74 (95% CI, 7.16–10.66) in comparison, 9.26 (7.54–11.39) in PID, and 28.73 (21.07–39.16) in endometriosis cohorts. The adjusted hazard ratio (aHR) of ovarian cancer was 1.17 (*p* = 0.296) in PID and 3.12 (*p* < 0.001) in endometriosis cohorts, compared with the comparison cohort in full cohort, using the multiple Cox regression model. The aHR of ovarian cancer was 0.83 (*p* = 0.650) in PID and 3.03 (*p* = 0.001) in endometriosis cohorts, compared with the comparison cohort after performing PSM. In the full cohort and PSM population, the cumulative incidence rate of ovarian cancer was significantly higher in patients with endometriosis than in those with PID or in the comparison cohort (*p* < 0.001 and *p* < 0.001). In conclusion, after considering the differences in the impacts of exposure to endometriosis or PID, patients with endometriosis were more likely to develop ovarian cancer.

## 1. Introduction

The age-standardized incidence of ovarian, fallopian tube, and broad ligament cancers was reported to be 9.18 per 100,000 women by the Taiwan Health Promotion Administration of the Ministry of Health and Welfare according to the annual cancer registry report in 2016. This group cancer, mainly consisting of ovarian cancer, ranks the seventh most common female malignancy and also the seventh mortality rate in females in this country. Ovarian cancer is known as one of the cancers with the worst prognosis in female individuals because it is often found in the advanced stages due to less obviously early symptoms.

It is estimated that chronic infection and inflammation lead to approximately 25% of cancer cases [1]. Inflammation could mediate and stimulate the production of tumor-promoting compounds, including cytokines, especially interleukin 6 and 8 and chemokines, as well as reactive oxygen species and lipid hydroperoxides [2,3,4]. The inflammatory mediators could eventually lead to a variety of DNA damages, genetic mutations and epigenetic changes and, thereafter, cause an increased susceptibility to the carcinogenic processes [1,5,6,7]. Pelvic inflammatory disease (PID) and endometriosis are very common gynecological pelvic inflammatory conditions. In the recent period, PID and endometriosis have aroused great research interests in the development of ovarian cancer. 

Endometriosis refers to the presence of endometrial tissue outside the uterus, and is presented as pelvic pain and infertility [8,9]. It has an impact on approximately 5–15% of women in the reproductive age and 3–5% of postmenopausal female individuals, and the various ethnic population differs greatly [8]. It has been reported that up to 1% of endometriosis cases have a malignant transformation [10]. In a large register study of 20,686 Swedish women who were admitted for endometriosis, the risk of developing ovarian cancer was elevated and the standardized incidence ratio during a mean follow-up of 11.4 years was 1.9 (95% confidence interval, 1.3–2.8) [11]. The most frequent genomic mutations in these carcinomas were explored to be mutations in the AT-rich interaction domain-containing protein 1 gene (ARID1A) [12]. On the contrary, it has been reported that women with endometriosis did not seem to be at increased risk of developing endometrial cancer based on a large prospective cohort of U.S. nurses [13]. PID is defined as an infection of the female upper genital tract, which is caused by microorganisms colonizing the vagina or endocervix and ascending to the endometrium and fallopian tubes [14,15]. It may affect the adjacent pelvic organs and then manifests as salpingitis, endometritis, a tubo-ovarian abscess, and pelvic peritonitis. From subtle or mild symptoms to severe sequelae of inflammation, such as pelvic pain and tubal factor infertility, the clinical manifestations vary greatly. It has been estimated that as many as 20% of Western women suffer from PID in their lifetime [16]. Previous results regarding whether PID increases the risk of ovarian cancer are inconsistent [17,18,19,20].

So far, there has not been any study comparing endometriosis and PID to assess which inflammatory diseases can increase the risk of ovarian cancer. It was inferred that the influences of endometriosis and PID on ovarian cancer are different. Therefore, it is necessary to conduct a large epidemiological study using a nationwide population cohort to simultaneously investigate the ovarian cancer risk of endometriosis and PID patients. The purposes of this study were to explore and compare the rate and risk of ovarian cancer in Taiwanese women with endometriosis or PID using a nationwide population-based design.

## 2. Materials and Methods

### 2.1. Research Design and Source of Data

A retrospective nationwide population cohort study was conducted to define the rate and risk of ovarian cancer in Taiwanese women with endometriosis or PID. These data were collected from the Longitudinal Generation Tracking Database 2000 (LGTD 2000) and the detailed information about LGTD can be obtained on the webpage [21]. LGTD 2000 included two million individuals randomly selected from National Health Insurance (NHI) Research Database (NHIRD) of Taiwan. NHI was established in 1995 and currently covers more than 99% of Taiwan’s population. LGTD 2000 is a representative subset of NHIRD data, with similar distributions of demographic data and NHI data in the healthcare field. In addition, the Taiwan Cancer Registry (TCR) dataset is a population-based cancer registry system launched in 1979 to obtain detailed information about cancer diagnosis, incidence, patient demographics, date of diagnosis, tumor location, histology, stage, and the evaluation of cancer control and prevention and prognosis [22]. Both LGTD 2000 and TCR are cross-linked and supervised by the Health and Welfare Data Science Center, Taiwan. The research used anonymous secondary data of LGTD 2000 and TCR. To waive the informed consent, approval was obtained from the Chung Shan Medical University Hospital Institutional Review Board (CSMUH No: CS17129).

### 2.2. Identification of Research Cohort and Inclusion and Exclusion Criteria

In total, LGTD 2000 enrolled 982,495 female individuals. The diagnoses of endometriosis and PID were separately determined based on the International Classification of Diseases (ICD), Ninth Revision, Clinical Modification (ICD-9-CM) code 617, and codes 614 and 615. We defined the cases with endometriosis or PID when the patients had ≥2 outpatient visits or any hospitalization associated with relevant ICD-9 cm codes. In total, 202,776 patients were diagnosed with endometriosis or PID while 779,719 individuals were never diagnosed as having endometriosis or PID.

The index date was determined as one year after the first date of the diagnosis of endometriosis or PID. The one-year lag period can control the surveillance bias caused by the utilization rate of health care. When the diagnoses of endometriosis and PID increased the frequency of medical examinations in that lag year, the probability of finding subclinical ovarian cancer also increased. In the identified endometriosis or PID population, the following subjects were excluded: (1) 49,922 females were diagnosed with endometriosis or PID before January 2002 or after December 2014 because the LGTD 2000 is the left-truncation [23] and right-censoring of the data; (2) 3343 patients diagnosed with any cancer (including ovarian cancer) before the index date; (3) 2252 female beneficiaries who were younger than 12 or older than 65 on the index date; (4) 7783 women who had undergone hysterectomy, oophorectomy, or salpingectomy; (5) 4240 women diagnosed with both endometriosis and PID simultaneously. After exclusion, the remaining female individuals without endometriosis and PID were matched with women with endometriosis or PID by age on the index date. On the index date, all females participating in the study were at risk. Finally, the research included 135,236 comparisons (general population without endometriosis and PID), 114,726 patients with PID, and 20,510 patients with endometriosis to assess the risk of ovarian cancer (full cohort).

### 2.3. Study Events and Covariates and Primary Outcomes

From the index date to the end of the study (31 December 2014), the TCR dataset was used to define the incidence of ovarian cancer for each study subject. The diagnosis of ovarian cancer was determined to be ICD-9 code 183.0 or ICD, Tenth Revision codes C56. In addition, the ICD-O-3 morphology code was included from the TCR. The risks of ovarian cancer were assessed for each study subject. In addition, comorbidities were recognized based on ICD-9 cm codes and were determined if there were at least two ICD-9 cm records for outpatient visits or one diagnostic code for hospitalization during the baseline period (within 1 year before the index date). The identifications of comorbidity were based on ICD-9 cm codes from LGTD 2000 as the followings: brain tumor, 191.x, 239.x; obesity, 278.x; renal disease, 580.x–589.x; hypertension, 401.x–405.x; diabetics mellitus, 250.x; lipid dysfunction, 272.x; ischemic heart disease, 410.x–414.x; ischemic stroke, 433.x–436.x; hyperthyroidism, 242.x; hypothyroidism, 244.x; chronic hepatitis, 571.4, 571.5, 571.6, 571.7, 571.8, 571.9, 573.1, 573.2, 573.3; chronic obstructive pulmonary disease, 491.x, 492.x, 496.x. The primary outcome was to compare the risk of ovarian cancer among patients with endometriosis, PID, and comparisons.

### 2.4. Propensity Score Matching

In order to reduce the unbalanced characteristics among endometriosis, PID, and comparison cohorts, propensity score matching (PSM) was performed as a sensitivity analysis to adjust the confounding effects. Conditional proportional hazard was used to assess the hazard ratios (HRs) of ovarian cancer in the PSM population in the sensitivity analysis [24]. The propensity score was the odds of endometriosis according to the demographic data, including birth year, age (±1 year) on the index date, index year, marital status, education level, and comorbidities. The nearest neighbor greedy algorithm was performed to match PID patients and comparison females with endometriosis patients, with 20,478 subjects in each cohort in a 1:1:1 ratio.

### 2.5. Statistical Analysis

The maximum of standardized mean difference (Max SMD) was calculated to check the balance of baseline characteristics and comorbidity variables in study cohorts, where the imbalance was defined as the percentage exceeded 10% [23,24]. It was defined as maximum standardized difference among standardized difference between comparison cohort and PID cohort, standardized difference between comparison cohort and endometriosis cohort, as well as standardized difference between PID cohort and endometriosis cohort.

The occurrence of ovarian cancer was assessed in the patients with endometriosis or PID, and the comparison cohorts. The follow-up started on the index date and ended on the date of confirmation of ovarian cancer, death, or 31 December 2014, whichever occurred first. Then, the follow-up interval was calculated as person–month. The incidence rate (per 100,000 person–years) and its 95% confidence interval (95% CI) of ovarian cancer were defined using Poisson regression. The Kaplan–Meier estimator was performed to define the cumulative incidence probability of ovarian cancer in the study cohorts.

The hazard ratios of ovarian cancer for patients with endometriosis or PID and the comparison cohort as a reference cohort were calculated and compared. Multiple Cox regression model was conducted to estimate the adjusted hazard ratio (aHR) of ovarian cancer in adjusting the covariates, including demographic variables and comorbidities. The aHR was estimated using the conditional Cox regression model in PID, endometriosis, and comparison cohorts after performing PSM [25,26,27]. Statistical significance was defined as *p* < 0.05. All statistical analyses were performed using SAS V. 9.4 software (SAS Institute Inc., Cary, NC, USA).

## 3. Results

### 3.1. Baseline Demographics and Comorbidity Characteristics in Full Cohort and Well Balanced in PSM Population

Overall, 135,236 age-matched comparisons, 114,726 patients with PID, and 20,510 patients with endometriosis were selected to assess the risk of ovarian cancer in a full cohort. Some differences were noted between patients with PID and endometriosis regarding the birth year and index year. Patients with endometriosis were more centralized in the birth years between 1963 and 1975. The endometriosis cohort was older than the PID cohort. As compared to PID patients and the comparison cohort, endometriosis patients had a higher proportion of comorbidities, including brain tumor, hypertension, lipid dysfunction, chronic hepatitis, and chronic obstructive pulmonary disease (Table 1). Thereafter, the aHR of ovarian cancer for patients with endometriosis or PID, and the comparison cohort as a reference cohort, was defined using multiple Cox regression model after adjusting these covariates.

After performing PSM, which was based on the odds of endometriosis, there were 20,478 female individuals in each study cohort. The matched cohorts were well balanced for baseline characteristics, and the Max SMD was less than 10% after performing PSM (Table 1). Then, the aHR was estimated using the conditional Cox regression model in PID and endometriosis patients.

### 3.2. Ovarian Cancer Risks and Rates among Research Cohorts in Full Cohort and PSM Population

During the entire observation period, the incidence proportion of ovarian cancer was 97 out of 1,109,986 (0.009%), 90 out of 971,419.2 (0.009%), and 40 out of 139,237.9 (0.029%) female individuals in the comparison, PID, and endometriosis cohorts, respectively. The incidence rate (per 100,000 person–years) of ovarian cancer was 8.74 (95% CI, 7.16–10.66) in comparison, 9.26 (7.54–11.39) in PID, and 28.73 (21.07–39.16) in endometriosis cohorts (Table 2). After adjusting for the covariates, including the year of index date, age on the index date, marital status, education level, and comorbidities in the multiple Cox regression, the aHR of ovarian cancer was 1.17 (95% CI, 0.87–1.56; *p* = 0.296) in the PID cohort and 3.12 (95% CI, 2.15–4.52; *p* < 0.001) in the endometriosis cohort, as compared to the comparison cohort (Table 2).

After performing PSM, the incidence proportion of ovarian cancer was 15 out of 139,146.2 (0.011%), 13 out of 140,138.7 (0.009%), and 40 out of 139,181.9 (0.029%) women in the comparison, PID, and endometriosis cohorts, respectively. The incidence rate (per 100,000 person–years) of ovarian cancer was 10.78 (95% CI, 6.50–17.88) in comparison, 9.28 (5.39–15.98) in PID, and 28.74 (21.08–39.18) in the endometriosis cohorts (Table 3). The aHR of ovarian cancer was 0.83 (95% CI, 0.38–1.79; *p* = 0.650) in the PID cohort and 3.03 (95% CI, 1.62–5.68; *p* = 0.001) in the endometriosis cohort, compared with the comparison cohort (Table 3).

### 3.3. Cumulative Incidence of Ovarian Cancer in Full Cohort and PSM Population

**Figure 1:**
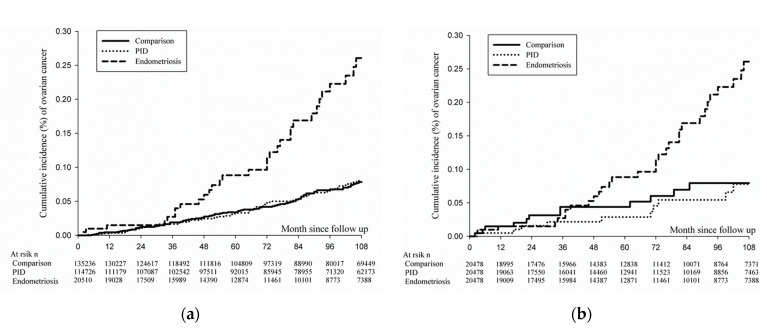
Kaplan–Meier curves for ovarian cancer among PID, endometriosis, and comparison cohorts. (**a**) Before (*p* < 0.001) and (**b**) after (*p* < 0.001) propensity score matching. PID, pelvic inflammatory disease.

The cumulative incidence rate of ovarian cancer in patients with endometriosis was significantly higher than that in PID or in comparison cohorts in the full cohort (*p* < 0.001; Figure 1a). It also indicated that the incidence rate of ovarian cancer in the endometriosis cohort was significantly higher after performing PSM, which could be regarded as a sensitivity analysis (*p* < 0.001; Figure 1b), as compared to that in PID or in comparison cohorts. Figure 1a indicates the cumulative incidence rates of ovarian cancer in the comparison, PID, and endometriosis cohorts, respectively in the full cohort. In the comparison cohort, the cumulative incidence rates were 0.012%, 0.028%, 0.042%, and 0.068% at 24, 48, 72, and 96 months after index date, respectively. In the PID cohort, the cumulative incidence rates were 0.011%, 0.025%, 0.048%, and 0.067%, respectively. In the endometriosis cohort, the cumulative incidence rates were 0.015%, 0.060%, 0.114%, and 0.223% at 24, 48, 72, and 96 months, respectively. Figure 1b indicates that after PSM, the cumulative incidence rate of ovarian cancer in patients with endometriosis was significantly higher than that in PID or in the comparison cohorts (*p* < 0.001).

## 4. Discussion

After considering the differences in the impacts of exposure to endometriosis or PID, patients with endometriosis were more likely to develop ovarian cancer. A Swedish Inpatient Register used nationwide population data to find a significantly increased ovarian cancer in patients with ovarian endometriosis (standardized incidence ratio (SIR), 1.92; 95% CI, 1.3–2.8) [28]. Based on a retrospective observational cohort study conducted in the United States, endometriosis patients had the highest risk with an SIR of 2.48 (95% CI, 1.3–4.2) compared with the general population [29]. Based on a survey conducted by the Shizuoka Cancer Registry, the SIR of ovarian cancer incidence in patients with endometriosis was 8.95 (95% CI, 4.12–15.3) [30]. However, SIR in these studies was estimated by univariate analysis. Based on a validation study in United States among Nurses’ Health Study II (NHSII), a significant association was found between endometriosis and ovarian cancer using multivariate Cox proportional hazard regression models [13]. In contrast, Olsen et al. did not reveal an increased ovarian cancer risk in endometriosis patients based on Iowa population [31]. Nomelini et al. also did not find a significant association between endometriosis and ovarian cancer according to the Hospital’s database [32]. Whereas a pooled analysis of 13 case–control studies of ovarian cancer in endometriosis women revealed an increased ovarian cancer risk [33]. The pooled analysis stemmed from all studies in the Ovarian Cancer Association Consortium (OCAC). The OCAC was founded in 2005 to foster collaborative efforts to discover and validate relationships between genetic polymorphisms and the risk of ovarian cancer. Data for endometriosis were investigated in 13 case–control studies of ovarian cancer. One study was undertaken in Australia, three in Europe, and nine in the USA. The studies of Olsen et al. and Nomelini et al. could not reveal a significant association between endometriosis and ovarian cancer. However, their study subjects restricted to the Iowa population and hospital-based patients wound limit external validity. In addition, record bias may exist because of an inaccurate self-reported endometriosis diagnosis in the Iowa women’s health study in Olsen et al.’s study, whereas the majority of investigated medical literatures state an increased risk of ovarian cancer among women with endometriosis [13,28,29,30,34,35]. These studies had a large population cohort or pooled analysis of a larger sample case–control study. However, to our knowledge, our study may be the largest epidemiological study using a nationwide population cohort to simultaneously investigate the ovarian cancer risk of endometriosis and PID patients by a multivariate analysis and find that patients with endometriosis are at risk of ovarian cancer by a full cohort and PSM population as a sensitivity analysis to validate our findings. A meta-analysis of the cohort and case–control studies suggested that endometriosis elevates the risk of ovarian cancer [36]. This meta-analysis was an electronic search performed to identify relevant studies published online since January 1990 to December 2012, 20 case–control and 15 cohort studies were recruited from 1625 potentially relevant studies.

Nevertheless, it has been reported that the risk in patients with endometriosis of developing ovarian cancer was higher than that in the general population. This is particularly true in considering the risk to develop the clear cell or endometrioid histotypes [37,38]. Moreover, it was reported that there were some evidences for shared genetic risks between endometriosis and all histotypes of ovarian cancer, except for the intestinal mucinous type [39]. Clear cell carcinoma exhibited the strongest genetic correlation with endometriosis (0.51, 95% CI = 0.18–0.84). Endometrioid carcinoma had moderate correlation with endometriosis. (0.48, 95% CI = 0.07–0.89). It displayed that shared genetic susceptibility mutations between endometriosis and ovarian cancer may lead to their epidemiological associations. The existence of the ARID1 mutations has been considered to be the most important genetic change in the malignant transformation of endometriosis [40]. Ishikawa et al. revealed a high frequency of ARID1A mutations in endometriosis-associated ovarian cancer [41]. ARID1A mutations often occur together with other concurrent mutations, resulting in the activation of the PI3K/Akt pathway [42]. Pavlidou et al. found the common genetic changes in endometriosis and ovarian cancer, stressing on the PI3K/Akt/mTOR signaling pathway [43]. In addition, KRAS mutations have been found in 29% of endometriosis-associated adenocarcinoma [44]. Furthermore, epigenetic modification is suggested to contribute not only to endometriosis, but also to the mechanisms of the malignant transformation of endometriosis such as DNA methylation and demethylation, histone modifications, and miRNA aberration by cumulative evidences [45].

Although this nationwide population cohort research revealed that patients with endometriosis exhibited a significantly increased risk of ovarian cancer, PID patients did not have a significantly increased risk of ovarian cancer by the full cohort and PSM population. Neutrophils can produce a large number of cytokines/chemokines, which are necessary for effector cell recruitment, activation, and response in inflammation [46]. Mononuclear phagocytes are guided to tissue injury sites by chemokines (such as monocyte chemoattractant protein-1, -2 and -3) and cytokines (such as interleukin-1β (IL-1β) and tumor necrosis factor-α (TNF-α)) [47]. It is important for the development of chronic disease when cytokines/chemokines persist in the sites of inflammation [47]. When the tissue regenerates during the injury process, it promotes cell proliferation, and after the assault agents are resolved, the proliferation and inflammation subside. Whereas proliferating cells that maintain DNA damage and/or mutagenic attacks continue to proliferate in microenvironments rich in inflammatory cells as well as growth/survival factors which nourish their growth, tumors occur when they are beyond comparisons [48]. Inflammatory mediators, such as TNF-α and IL-1β, are known as NF-κB activators [49]. After its release, NF-κB may stimulate malignancy-promoting signaling pathways in both cancer cells and cancer-related inflammatory cells [50]. It has been reported that chronic inflammation enhances the malignant transformation of ovarian epithelial cells [51]. Considering it as a possible explanation of the pathological relationship between endometriosis and ovarian cancer and possible causal mechanisms, there were differences between endometriosis and PID to explain why PID does not increase risk. It could be partly explained by the fact that PID patients may have been cured, and the duration of inflammation was not as long as that of endometriosis. It can also be partly attributed to the fact that the severity of inflammation in PID patients is lower than that in endometriosis patients.

Epidemiological studies have revealed a conflicting association between PID and ovarian cancer risk. Lin et al. suggested that the adjusted hazard ratio of ovarian cancer in patients with PID was 1.92 (95% CI, 1.27–2.92) as compared to comparisons using a population-based design [52]. Piao et al. indicated that PID was related to an increased risk of ovarian cancer using a meta-analysis [53]. Zhou et al. found that PID may be a potential risk factor for ovarian cancer, and there is an obvious association among Asian women [54]. In contrast, Parazzini et al. excluded an increased risk of ovarian cancer in PID patients based on the limited numbers of a case and control research [55]. Rasmussen et al. pooled data from 13 case–control studies and reported that no association was found between PID and ovarian cancer risk [56]. Shen et al. demonstrated that no increased risk for ovarian cancer was found in PID patients compared with a matching population by a nationwide population-based cohort study [57]. Our research is the first study to simultaneously assess the impacts of PID and endometriosis on the development of ovarian cancer, and find that PID patients do not have a significantly increased risk of ovarian cancer. Among the researches about PID patients increasing the risk of ovarian cancer, the time was too short in assessing the risk of developing ovarian cancer up to 3 years follow-up after PID in the study of Lin et al. In the meta-analysis study of Zhou et al., who found that PID was related to an increased risk of ovarian cancer, the heterogeneity was high (I^2^ = 58.8%). In the meta-analysis study of Piao et al., there was moderate heterogeneity (I^2^ = 41.0%) across studies in the initial analysis. After stratified by race, a significant positive association was observed in studies among Asian women (relative risk, 1.69, 95% CI, 1.22–2.34; I^2^ = 0%). Among the researches about there not being an increased risk of ovarian cancer in PID patients, the sample size of the study of Parazzini et al. was limited. The pooled data analysis based on 13 case–control studies also showed no relationship between them in the study of Rasmussen et al. Nevertheless, case–control studies lacked the cause–effect relationship. In agreement with our findings, Shen et al. found no association between PID and ovarian cancer. However, the follow-up duration (median: 8.84 years) might be insufficient for assessing the ovarian cancer risk. However, our study used a nationwide population cohort study from Taiwanese women with a sufficiently long period (2000 to 2014) and enough sample size dataset, and adjusted several possible confounding factors and performed PSM, which could be used as a sensitivity analysis for a full cohort and further validating the results of this research. Based on clinicopathological findings, endometriosis has been reported to be the precursor of ovarian clear cell carcinoma and ovarian endometrioid carcinoma, which are, therefore, termed as “endometriosis-associated ovarian carcinoma” [33]. Whole-exome sequencing has explored that 79% of patients with deep-infiltrating endometriosis had some harboring oncogenic driver mutations, such as ARID1A, KRAS, PIK3CA, and PPP2R1A [58]. Corresponding to pathological characteristics, atypical endometriosis is known as a precursor lesion of clear cell carcinoma or endometrioid carcinoma at the genomic level [59,60]. These could explain the significant relationship between ovarian cancer and endometriosis but not PID.

This study has some strengths. A first nationwide population cohort study from Taiwanese women was enrolled from a sufficiently long period (2000 to 2014) dataset, which allowed the authors to track and compare the subsequent risks of ovarian cancers in the patients with endometriosis, those with PID, and comparison cohort. This can prevent the results from being biased due to left-truncation and left-censoring information [61], especially when the short-time window is applied [52]. This study adjusted several possible confounding factors and performed PSM, which could be used as a sensitivity analysis for a full cohort and further validating the results of this research due to similar findings. In addition, the index date was lagged by one year to account for bias caused by an increase in healthcare utilization within one year after the diagnosis of PID or endometriosis. The study also had some limitations. Fewer ovarian cancer cases could be recruited for a statistical analysis after performing PSM because of a low incidence of the diseases themselves in Taiwan. Another limitation was that the histologic types of ovarian cancer such as clear cell carcinoma and endometrioid carcinoma could not be further subclassified for a statistical analysis because of limited samples. A surveillance bias might exist; however, it was resolved by lagging the index date as one year after the diagnosis of PID or endometriosis [62]. Another limitation was that this study lacked some explanations and clarity around how the results of this study related to the topics discussed (e.g., mutations). The mutations as well as the changes of cytokines/chemokines, molecular factors, and signaling pathways related to endometriosis, PID and ovarian cancer need their samples to analyze if there are similar mutations or molecular changes among endometriosis, PID, and ovarian cancer in an experimental study in the future. The inferred relationships among them were based on previous studies mentioned above. The translational significance of the information, including our epidemiological results as well as probable molecular and genetic data derived from previous and future experimental studies, may provide targeted screening to women most at risk for ovarian cancer. Therefore, Taiwanese patients with endometriosis have these epidemiological characteristics and oncogenic mutations should be intensely followed and treated to prevent the occurrence of ovarian cancer.

## 5. Conclusions

In conclusion, our research showed that patients with endometriosis had a significantly higher ovarian cancer risk than did the comparisons in the full cohort and the PSM population. After considering the differences in the impacts of exposure to endometriosis or PID, patients with endometriosis were more likely to develop ovarian cancer but those with PID were not.

## Figures and Tables

**Table 1:** Baseline characteristics of exposure (PID or endometriosis) and comparison cohorts with age and birth year matched and after performing PSM.

	No. (%)				
Characteristics	Comparisons	PID	Endometriosis	Max SMD (Before PSM)	Max SMD (After PSM) *
No. in cohort	135,236	114,726	20,510		
Birth year				32.95%	8.56%
1937–1949	3474 (2.57%)	3274 (2.85%)	200 (0.98%)		
1950–1962	29,272 (21.65%)	23,653 (20.62%)	5619 (27.40%)		
1963–1975	49,587 (36.67%)	40,605 (35.39%)	8982 (43.79%)		
1976–1988	46,103 (34.09%)	41,117 (35.84%)	4986 (24.31%)		
1989–2002	6800 (5.03%)	6077 (5.30%)	723 (3.53%)		
Year of index				45.67%	2.39%
2002–2006	80,807 (59.75%)	71,948 (62.71%)	8859 (43.19%)		
2007–2010	31,435 (23.24%)	25,854 (22.54%)	5581 (27.21%)		
2011–2014	22,994 (17.00%)	16,924 (14.75%)	6070 (29.60%)		
Age at index date				50.14%	0.00%
12–25	29,606 (21.89%)	27,304 (23.8%)	2302 (11.22%)		
26–35	43,757 (32.36%)	38,537 (33.59%)	5220 (25.45%)		
36–45	36,730 (27.16%)	29,119 (25.38%)	7611 (37.11%)		
46–55	21,304 (15.75%)	16,168 (14.09%)	5136 (25.04%)		
56–65	3839 (2.84%)	3598 (3.14%)	241 (1.18%)		
Marital status				21.24%	4.03%
Single	59,712 (44.15%)	39,212 (34.18%)	7096 (34.60%)		
Married	65,558 (48.48%)	63,066 (54.97%)	11,873 (57.89%)		
Others	9966 (7.37%)	12,448 (10.85%)	1541 (7.51%)		
Education level (years)				27.58%	2.47%
<7	25,079 (18.54%)	23,337 (20.34%)	2792 (13.61%)		
7–9	25,627 (18.95%)	25,655 (22.36%)	3697 (18.03%)		
10–12	52,137 (38.55%)	47,233 (41.17%)	8323 (40.58%)		
≥13	32,393 (23.95%)	18,501 (16.13%)	5698 (27.78%)		
Comorbidities					
Brain tumor	666 (0.49%)	787 (0.69%)	326 (1.59%)	10.82%	1.94%
Obesity	489 (0.36%)	499 (0.43%)	145 (0.71%)	4.74%	1.15%
Renal disease	1036 (0.77%)	1303 (1.14%)	298 (1.45%)	6.56%	1.60%
Hypertension	5037 (3.72%)	5016 (4.37%)	1359 (6.63%)	13.13%	0.34%
Diabetics mellitus	2682 (1.98%)	2959 (2.58%)	712 (3.47%)	9.15%	2.09%
Lipid dysfunction	4183 (3.09%)	4749 (4.14%)	1273 (6.21%)	14.83%	1.84%
ISH	1317 (0.97%)	1912 (1.67%)	409 (1.99%)	8.45%	1.11%
Ischemic stroke	358 (0.26%)	369 (0.32%)	105 (0.51%)	3.98%	0.56%
Hyperthyroidism	1591 (1.18%)	1925 (1.68%)	431 (2.10%)	7.29%	0.62%
Hypothyroidism	511 (0.38%)	597 (0.52%)	227 (1.11%)	8.50%	1.82%
Chronic hepatitis	4200 (3.11%)	5528 (4.82%)	1361 (6.64%)	16.46%	0.73%
COPD	3949 (2.92%)	4651 (4.05%)	1034 (5.04%)	10.87%	0.00%

* 20,478 subjects in each cohort after performing PSM; PID, pelvic inflammatory disease; PSM, propensity score matching; ISH, ischemic heart disease; COPD, chronic obstructive pulmonary disease; Max SMD, maximum of standardized mean difference, the imbalance characteristic was determined with the Max SMD >10%.

**Table 2:** Incidence rate and risk of ovarian cancer before PSM (age and birth year matched between exposure (PID/endometriosis) and comparison cohorts).

	Comparisons	Exposure (PID)	Exposure (Endometriosis)
N	135,236	114,726	20,510
Ovarian cancer			
Follow up person–years	1,109,986	971,419.2	139,237.9
Event	97	90	40
Rate ^‡^ (95% CI)	8.74 (7.16–10.66)	9.26 (7.54–11.39)	28.73 (21.07–39.16)
aHR * (95% CI)	Reference	1.17 (0.87–1.56)	3.12 (2.15–4.52)

^‡^ Crude rate, per 100,000 person–years. * aHR: adjusted hazard before PSM, multiple Cox regression was performed to adjust the covariates, including year of index, age at index date, marital status, education level, and comorbidities. PID, pelvic inflammatory disease; PSM, propensity score matching.

**Table 3:** Incidence rate and risk of ovarian cancer after performing PSM.

	Comparisons	Exposure (PID)	Exposure (Endometriosis)
N	20,478	20,478	20,478
Ovarian cancer			
Follow up person–years	139,146.2	140,138.7	139,181.9
Event	15	13	40
Rate ^‡^ (95% CI)	10.78 (6.50–17.88)	9.28 (5.39–15.98)	28.74 (21.08–39.18)
aHR * (95% CI)	Reference	0.83 (0.38–1.79)	3.03 (1.62–5.68)

^‡^ Crude rate, per 100,000 person–years. * aHR: adjusted hazard ratio after PSM, estimated using the conditional Cox regression model. PID, pelvic inflammatory disease; PSM, propensity score matching.

## Data Availability

Restrictions apply to the availability of these data. Data were obtained from the National Health Insurance database and are available from the authors with the permission of the National Health Insurance Administration of Taiwan.
